# Plasma brain natriuretic peptide is a biomarker for screening ischemic cerebral small vessel disease in patients with hypertension

**DOI:** 10.1097/MD.0000000000012088

**Published:** 2018-08-21

**Authors:** Weimin Wei, Yan Chen, Da Lei, Yanan Zhang, Xiuhong Weng, Yuliang Zhou, Li Zhang

**Affiliations:** a Department of Cardiology, The First Affiliated Hospital of Guangdong Pharmaceutical University; b Department of Neurology, Zengcheng People's Hospital (Boji-Affiliated Hospital of Sun Yat-sen University); c School of Clinical Medicine, The First Affiliated Hospital of Guangdong Pharmaceutical University, Guangzhou; d Veterinary medicine, Northeast Agricultural University, Haerbin, China.

**Keywords:** brain natriuretic peptide, cerebral small vessel disease, ischemia, magnetic resonance imaging

## Abstract

Plasma brain natriuretic peptide (BNP), a diagnostic marker of cardiovascular diseases, has been previously linked to cerebrovascular diseases. Our goal was to determine whether plasma BNP level is helpful for identifying high-risk individuals who are likely to present with the 3 main subtypes of cerebral small vessel diseases (CSVDs), namely, white matter lesions, lacunar infarcts, and cerebral microbleeds, on magnetic resonance imaging (MRI) in patients with hypertension.

Three hundred forty-six consecutive hypertensive patients presenting at our cardiology or neurology clinic were investigated. Plasma BNP level was measured by chemiluminescent microparticle immunoassay. The presence of CSVD was assessed by 1.5-T brain MRI. Multivariate linear regression was used to determine whether individual or combined MRI-defined CSVD subtypes were associated with BNP level, after adjustment for several covariates.

The mean age of patients was 69.1 ± 9.8 years, and 44.2% were female. The highest quartile BNP group was positively associated with advanced age, female sex, clinically manifesting cardiac diseases, and ischemic CSVD (white matter lesions and lacunar infarcts) and no association with cerebral microbleeds. According to multivariate linear regression, white matter lesions [β = 0.722; 95% confidence interval (95% CI), 0.624–0.819] and lacunar infarcts (β = 0.635; 95% CI, 0.508–0.762) were independently associated with BNP level, even after controlling for vascular risk factors and clinically manifesting cardiac diseases. Combined white matter lesions and lacunar infarcts were more strongly associated with BNP level than each subtype alone. With the cutoff value of 106.4 pg/mL, BNP level had a sensitivity, a specificity, and an area under the curve of 95.2%, 64.9%, and 0.799, respectively, for white matter lesions, whereas the values were 143.0 pg/mL, 81.6%, 73.5%, and 0.848, respectively, for lacunar infarcts.

Plasma BNP level, which is independently correlated with individual or combined white matter lesions and lacunar infarcts, is a useful molecular marker for identifying ischemic CSVD in patients with hypertension.

## 1 Introduction

Cerebral small vessel disease (CSVD) is a neuropathological disorder characterized by abnormalities of small perforating arteries, arterioles, capillaries, and venules in the brain.^[1,2]^ CSVD contributes to 45% of all dementia cases^[3,4]^ and accounts for approximately 20% of all stroke cases.^[3–6]^ Functional disability and cognitive decline are also frequently seen in patients with CSVD.^[3,5,7,8]^ The pathogenesis of CSVD is poorly understood, and vascular risk factors have been implicated. Among them, hypertension is a major risk factor for the occurrence and development of CSVD.^[1,9]^ Hence, the early detection of subclinical brain damage caused by CSVD in patients with hypertension is important. Magnetic resonance imaging (MRI) is a validated technique that is used to detect the 3 main subtypes of CSVD, namely, white matter lesions, lacunar infarcts, and cerebral microbleeds.^[1,10,11]^ White matter lesions and lacunar infarcts can be called ischemic CSVD.^[10]^ In most cases, these MRI-defined CSVD, however, do not cause abrupt clinical symptoms. Hence, it should be feasible to identify high-risk individuals to perform brain MRI assessment.

Brain natriuretic peptide (BNP) is an active hormone that is primarily released from cardiomyocytes in response to volume overload and neurohumoral factors, whereas N-terminal pro-BNP (NT-proBNP) is an inactive fragment of prohormone proBNP. BNP and NT-proBNP may be used as practical biomarkers due to their rapid measurement through blood tests and can be helpful in the detection of disease at an early stage before structural and functional changes arise.^[12]^ Although both have good clinical performance, because of greater dependence on renal function for clearance and lower susceptibility to rapid change in levels due to the longer half-life, NT-proBNP has lower specificity for diagnosis than does BNP.^[13,14]^ Interestingly, increased levels of NT-proBNP are associated with subclinical cerebrovascular lesions and could be a marker of silent vascular brain injury in hypertension.^[5]^ However, whether or not BNP level could be considered a useful marker for identifying CSVD has rarely been reported or has contradictory results with NT-proBNP.^[8,15]^

Patients with CSVD should be closely monitored due to their generally poor outcomes. Therefore, the use of plasma BNP to identify high-risk individuals to be screened by brain MRI should be considered in hypertensive patients without overt neurological symptoms. In the present study, we hypothesize that individual or combined MRI-defined CSVD subtypes might be associated with plasma BNP level, and that these associations are independent of other vascular risk factors and clinically manifesting cardiac diseases. Moreover, we address whether plasma BNP level is a useful tool for clinicians in screening CSVD at an early stage in patients with hypertension.

## 2 Methods

### 2.1 Study population

We recruited consecutive patients with a diagnosis of essential hypertension at our cardiology or neurology outpatient clinic between January 2012 and December 2015. A total of 346 hypertensive patients (193 men and 153 women; mean age 69.1 ± 9.8 years) were enrolled in our study. Hypertension was defined as the systolic and/or diastolic blood pressure measurement consistently higher than 140/90 mm Hg or the use of antihypertensive medication in individuals aged 18 years or older. Hypertension duration was defined as the time from diagnosis to neuroimaging operation. The history of clinically manifesting diseases was verified from medical records or face-to-face interview based on data on hypertension duration, classification of blood pressure (hypertension stage 1 and stage 2 according to the guidelines ^[16]^), the type of antihypertensive medication, the history of diabetes and cardiac diseases (atrial fibrillation, ischemic heart disease, and heart failure), and the history of smoking status. Patients with acute cardiac decomposition, established cerebrovascular diseases, dementia, renal impairment [serum creatinine (Scr) level > 2.5 mg/dL)], pulmonary disease, including chronic obstructive pulmonary disease, pneumocardial disease and pulmonary embolism, and contraindication to MRI, were excluded from the study. Clinical data on blood pressure, body weight, and height of the patients were recorded during the day of MRI. Body mass index (BMI) was calculated using the patient's body weight in kilograms divided by the square root of the height in centimeters.

All patients provided written informed consents, and this project was approved by the Medical Ethics Committee of The First Affiliated Hospital/School of Clinical Medicine of Guangdong Pharmaceutical University, Guangzhou, China.

### 2.2 Blood test

Plasma BNP levels were measured by chemiluminescent microparticle immunoassay (CMIA) using ARCHITECT i2000 system and ARCHITECT BNP reagent kits 8K28 (Abbott Laboratories, Abbott Park, Illinois). The coefficient of variation for the BNP assay was less than 12%, and the analytic measurement range for BNP was 10 to 5000 pg/mL. At the same time, fasting blood glucose, triglyceride (TG), total cholesterol (TC), high-density lipoprotein cholesterol (HDL-C), low-density lipoprotein cholesterol (LDL-C), blood urea nitrogen (BUN), Scr, uric acid (UA) levels were detected. Then, estimated glomerular filtration rate (eGFR) was calculated according to the formula eGFR (mL/min/1.73 m^2^) = 30,849 × standardized Scr^−1.154^ × age^−0.203^ × 0.742 (if female).^[17]^

### 2.3 Magnetic resonance imaging (MRI)

Brain MRI was performed using a 1.5-Tesla MRI scanner (Signa HDxt; GE Healthcare, Milwaukee, Wisconsin) with an eight-channel phased-array head coil. For the evaluation of CSVD, a T1-weighted image, a T2∗-weighted image, and a fluid-attenuated inversion recovery (FLAIR) sequence were assessed. CSVD was defined as the presence of white matter lesions, lacunar infarcts, and cerebral microbleeds. White matter lesions were defined as neuroimaging abnormalities of the white matter with extensive or focal lesions that appeared as hyperintensity on T2-weighted or FLAIR images without mass effect.^[5,18]^ Subcortical and periventricular white matter lesions were evaluated together. Lacunar infarcts were defined as focal lesions ≥3 and <15 mm in size with similar signal intensity to that of cerebrospinal fluid on all pulse sequences, with a hyperintense rim on the FLAIR sequence when they were located supratentorially. Microbleeds were defined as focal areas of very low signal intensity on T2∗-weighted imaging that are not accompanied by evident signal abnormality on other structural sequences.^[19]^ All images were assessed primarily by 2 trained readers at our MRI Reading Center.

### 2.4 Statistical analysis

Statistical analyses were carried out using Stata 14.2 (Stata Corp., College Station, Texas). For continuous variables, data were expressed as the mean ± standard deviation (SD), and data for categorical variables were represented as frequencies and percentages. A Chi-square test was used for group comparisons of categorical variables, and analysis of variance (ANOVA) or the Kruskal–Wallis test was performed for continuous variables or continuous skewed variables. Plasma BNP level was not normally distributed and was log-transformed for statistical analysis. The association of vascular risk factors with log-BNP was analyzed using univariable linear regression for categorical and continuous variables. Coefficients (β) and *P* values were reported. To examine the association of individual or combined MRI-defined CSVD subtypes with log-BNP, a multivariable linear regression was performed. Coefficients (β), 95% confidence intervals (95% CIs), and *P* values were reported. Receiver operating characteristic (ROC) curve analysis was used to determine the cutoff plasma BNP values for the diagnosis of white matter lesions and lacunar infarcts. Two-sided *P* < .05 was considered statistically significant.

## 3 Results

### 3.1 Baseline characteristics of patients

A total of 346 hypertensive patients were recruited in the current study. Baseline characteristics of patients are summarized in Table 1. The mean hypertension duration in these patients was 9.6 ± 8.6 years. Among these hypertensive patients, 284 (82.1%) were diagnosed with CSVD (182 white matter lesions, 96 lacunar infarcts, and 132 cerebral microbleeds).

**Table 1:**
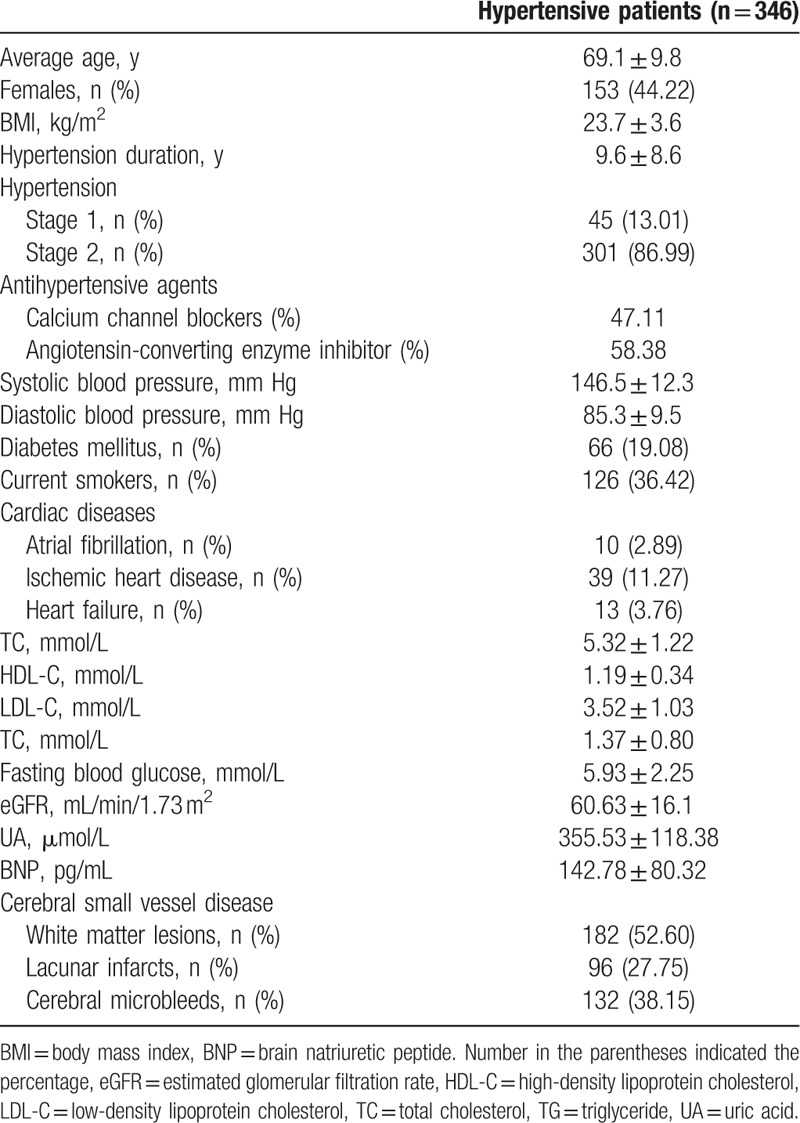
Baseline characteristics of hypertensive patients.

### 3.2 Clinical characteristics depending on quartiles of plasma BNP level

According to the quartiles of plasma BNP level, patients were classified into 4 groups, namely, <83.2 pg/mL, 83.2 pg/mL through 113.1 pg/mL, 113.1 pg/mL through 189.0 pg/mL, and >189.0 pg/mL (Table 2). Advanced age, female sex, atrial fibrillation, ischemic heart disease, heart failure, and lacunar infarcts were more prevalent in the highest quartile group than in the other groups (all *P* < .05). Serum TC and LDL-C levels were higher, and white matter lesions were more prevalent in the highest 2 quartile groups than in the other groups (all *P* < .05). However, serum HDL-C and eGFR were lower in the highest quartile group than in the other groups (all *P* < .05). The prevalence of cerebral microbleeds was not different among 4 groups (*P* > .05).

**Table 2:**
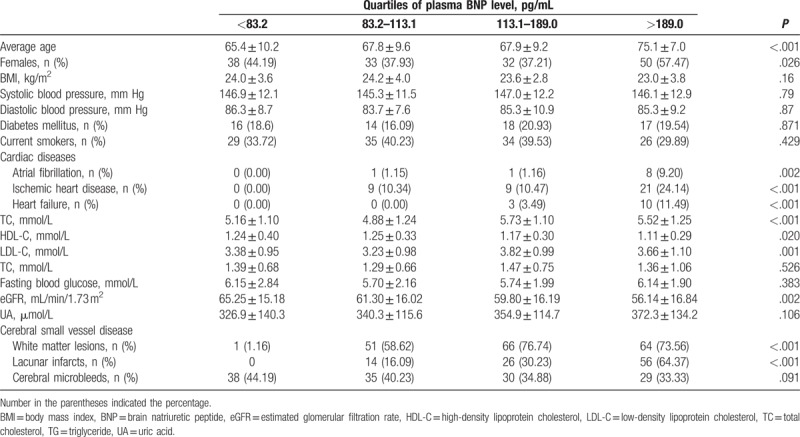
Clinical characteristics according to quartiles of plasma BNP level.

### 3.3 Association of individual or combined MRI-defined CSVD subtypes with BNP

The associations of CSVD with log-BNP level are summarized in Table 3. White matter lesions were significantly related to high level of log-BNP in age- and sex-adjusted model (β = 0.678; 95% CI, 0.565–0.791). After additional adjustments with vascular risk factors and clinically manifesting cardiac diseases, this association remained significant (β = 0.722; 95% CI, 0.624–0.819). Similarly, a significant association was also observed between lacunar infarcts and log-BNP in a multivariable adjusted model (β = 0.635; 95% CI, 0.508–0.762). For cerebral microbleeds, a negative correlation was observed with log-BNP (β = −0.132; 95% CI, −0.263 to 0.001; *P* = .047). However, this association disappeared after adjustment for vascular risk factors and clinically manifesting cardiac diseases. Vascular risk factors included BMI, serum TC, LDL-C and HDL-C level, and eGFR. These factors were verified to be associated with log-BNP level (*P* < .05, data not shown).

**Table 3:**
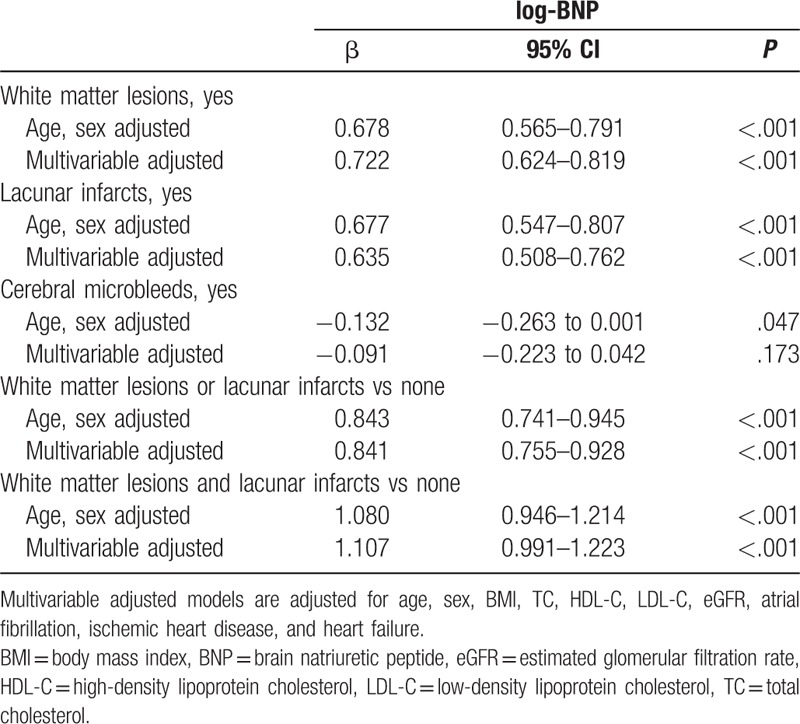
Association between individual or combined CSVD subtypes and BNP level.

The above results showed that ischemic CSVD (white matter lesions and lacunar infarcts) was associated with BNP level. Thus, we next analyzed the correlation between increasing severity of ischemic CSVD (combined white matter lesions and lacunar infarcts) and log-BNP (Table 3). Combined white matter lesions and lacunar infarcts were more strongly associated with log-BNP than each subtype alone in age- and sex-adjusted models [(β = 1.080; 95% CI, 0.946–1.214) vs (β = 0.843; 95% CI, 0.741–0.945)] or in the additional multivariable adjusted model [(β = 1.107; 95% CI, 0.991–1.223) vs (β = 0.841; 95% CI, 0.755–0.928)].

### 3.4 Cutoff values of BNP for predicting ischemic CSVD

**Figure 1:**
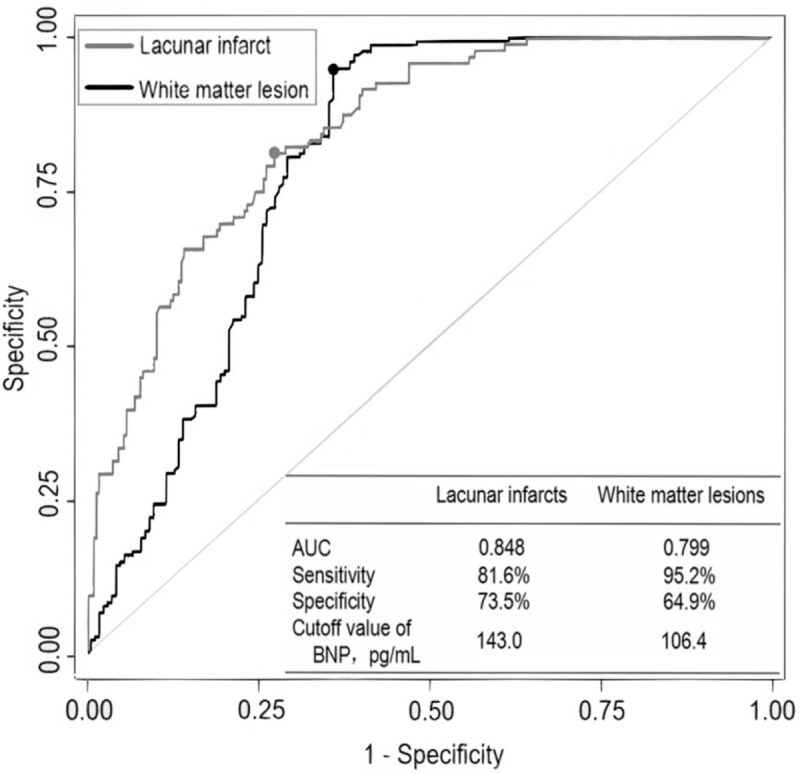
ROC curve of BNP level in predicting lacunar infarcts and white matter lesions. AUC = area under the curve, ROC = receiver operation characteristic.

Figure 1 shows the ROC curve on the basis of plasma BNP for the prediction of ischemic CSVD. ROC analysis of plasma BNP level and white matter lesions revealed an area under the curve (AUC) of 0.799 and a cutoff value of ≥106.4 pg/mL (sensitivity 95.2%; specificity 64.9%). ROC analysis of plasma BNP and lacunar infarcts revealed an AUC of 0.848 and a cutoff value of ≥143.0 pg/mL (sensitivity 81.6%; specificity 73.5%).

## 4 Discussion

In this population-based study, ischemic CSVD, including white matter lesions and lacunar infarcts, in patients with hypertension, was found to be related to increased plasma BNP level, independent of other vascular risk factors and clinically manifesting cardiac diseases. Furthermore, the association of combined white matter lesions and lacunar infarcts with plasma BNP level was stronger than that of each subtype alone. In addition, plasma BNP level was helpful for screening ischemic CSVD in hypertensive patients with the cutoff value of 106.4 and 143.0 pg/mL for white matter lesions and lacunar infarcts, respectively. These findings suggest that plasma BNP level may serve as a useful biomarker for screening ischemic CSVD in patients with hypertension.

Hypertension is the most prevalent modifiable vascular risk factor for CSVD.^[1,18]^ Patients with hypertension are at an increased risk of suffering from adverse effects on vasculature and end organs, with subsequent progressive CSVD. This notion may be supported with the hypothesis that sustained elevation and fluctuation of blood pressure in cerebral small vessels can result in microvascular damage.^[6,20]^ Studies have shown that the proportion of CSVD was higher in Chinese populations than in western populations.^[18,21]^ According to our study, CSVD accounted for 82.1% of all hypertension cases. In addition to hypertension, other risk factors for CSVD including age and diabetes mellitus have been established.^[1,18]^ Older age (69.1 ± 9.8 years) and longer hypertension duration (9.6 ± 8.6 years) may be attributed to the higher proportion of CSVD observed in this study. Among the subtypes of CSVD, white matter lesions were more prevalent than others, and these results are broadly consistent with the results of previous studies.^[6,22,23]^

Our study linked BNP to the subtype of CSVD. To the best of our knowledge, there are few studies evaluating the association of BNP with several markers of CSVD, either individually or combined. Pikula et al^[8]^ found no association between BNP and white matter lesions or lacunar infarcts when they were combined with other biomarkers such as inflammation, hemostasis, neurohormonal activity, and endothelial function. In another study, BNP concentrations correlated with atrial fibrillation-associated silent white matter lesions.^[15]^ However, the relationship between NT-proBNP and the subtype of CSVD has been discussed in some studies. In a population-based study of community-dwelling middle-aged and elderly subjects without dementia and clinical cardiovascular disease, higher serum NT-proBNP level was associated with larger white matter lesion volume.^[24]^ Significantly higher NT-proBNP level was identified in patients with cortical cerebral microinfarcts (CMIs) and found to be associated with an increasing number of CMIs.^[25]^ Others have also suggested that NT-proBNP is independently associated with silent lacunar infarcts and white matter lesions,^[5,26]^ as well as brain microbleeds, basal ganglia enlarged perivascular spaces and combined MRI markers of CSVD in fully adjusted models (vascular risks, previous heart diseases, including coronary events and heart failure, and atrial fibrillation).^[5]^ The authors also found that the association between NT-proBNP and brain microbleeds disappeared when patients with prior heart disease were excluded, ^[5]^ and these results are consistent with the findings of our study, although different analyses were used.

Our results support the hypothesis that specific biomarkers could be a useful tool for the early identification of subclinical brain diseases, mainly ischemic CSVD (lacunar infarcts and white matter lesions). We identified BNP level ≥106.4 and ≥143.0 pg/mL as valuable predictors for white matter lesions and lacunar infarcts, respectively. Plasma BNP as a biomarker may be a better predictor for lacunar infarcts than for white matter lesions, reaching an accuracy of nearly 90%. The explanation for this result is that the increase in BNP level could be more related to blood stasis, which is a well-known condition for thrombi formation.^[27]^

In addition to cardiovascular diseases, several reasons might explain the role of elevated plasma BNP level in brain vessel damage. First, BNP can augment cerebral blood flow reduction and ischemia damage by reducing local blood volume and blood pressure.^[28]^ We showed that elevated plasma BNP level is associated with ischemic CSVD in an adjusted model, suggesting that ischemic damage in the brain could be a potential source of circulating BNP independent of heart disease.^[29]^ Second, higher level of BNP and CSVD might share vascular risk factors, such as aging, diabetes, dyslipidemia, and hypertension, which can cause arteriolosclerosis and lead to clinical or subclinical cerebral injury. In our study, the association between subclinical ischemic CSVD and BNP level might be more driven by hypertension than other risk factors because all of our patients were hypertensive and because hypertension is commonly related to ventricular wall stress and subclinical brain damage.^[5]^

There are several limitations in our study. First, although this study consisted of randomly selected hypertensive patients, the proportion of CSVD was overrepresented, especially cases with cerebral microbleeds, and this might lead to errors in the association between MRI markers and BNP level. Second, volumetric measurements and classification of different locations of white matter lesions were not performed in this study. However, we considered subcortical and periventricular white matter lesions together. Third, 3 MRI markers of CSVD were observed in our study, and others such as enlarged perivascular spaces and cortical atrophy should be discussed in future studies. Fourth, a measure of cardiac function was not obtained at the time of MRI, although our results were validated in an adjusted model of clinically manifesting cardiac diseases.

Our findings suggest that individual or combined white matter lesions and lacunar infarcts are independently related to plasma BNP level in patients with hypertension. BNP level is useful for the early identifying individuals at a high-risk ischemic CSVD, especially lacunar infarcts. These results might be helpful for screening individuals who are likely to present with ischemic CSVD.

## Author contributions

**Conceptualization:** Weimin Wei, Da Lei, Li Zhang.

**Data curation:** Weimin Wei, Yanan Zhang, Li Zhang.

**Formal analysis:** Weimin Wei, Yan Chen, Xiuhong Weng.

**Methodology:** Weimin Wei, Yuliang Zhou.

**Project administration:** Yan Chen, Yanan Zhang.

**Writing – original draft:** Yan Chen.

**Writing – review & editing:** Li Zhang.
